# Association between fungal growth temperature and corneal lesions in fungal keratitis: a case series

**DOI:** 10.1186/s12886-026-05098-9

**Published:** 2026-07-06

**Authors:** Hidenori Inoue, Ayame Hyodo, Kano Tabata, Natsuki Kubota, Miyako Iyoda, Hitoshi Miyamoto, Shinobu Murakami, Koji Toriyama, Takashi Suzuki, Takashi Yaguchi, Atsushi Shiraishi

**Affiliations:** 1 Department of Ophthalmology, Ehime University Graduate School of Medicine, Shitsukawa, Toon, Ehime 791-0295 Japan; 2 Clinical Laboratory Division, Ehime University Hospital, Shitsukawa, Toon, Ehime 791-0295 Japan; 3 Department of Ophthalmology, Toho University Graduate School of Medicine, Omori-Nishi, Ota-Ku, Tokyo, 6-11-1 Japan; 4 Medical Mycology Research Center, Chiba University, 1-8-1, Inohana, Chuo-Ku, Chiba, 260-8673 Japan

**Keywords:** Fungal keratitis, Growth temperature, Corneal infiltration

## Abstract

**Background:**

Fungal keratitis is a refractory disease, and cases caused by filamentous fungi are particularly challenging to manage. Considering that the depth of corneal infiltration influences both treatment and visual prognosis, the ability of causative fungi to grow at corneal and anterior chamber temperatures was hypothesized to contribute to the progression of lesions from the deep cornea into the anterior chamber. This study investigated the relationship between the growth temperature of filamentous fungi isolated from keratitis and the depth of corneal infiltration.

**Methods:**

Eight cases of filamentous fungal keratitis diagnosed at Ehime University Hospital between January 2020 and April 2024 were retrospectively reviewed. Causative organisms were identified from corneal scrapings via morphological analysis, MALDI-TOF mass spectrometry, or sequencing of the internal transcribed spacer region. Growth temperature testing was performed on Sabouraud agar at 28 °C, 35 °C, and 37 °C, assessing colony formation after 5 days. The depth of corneal infiltration was clinically assessed, and its relationship with fungal growth temperatures was analyzed.

**Results:**

The causative fungi were Fusarium spp. (*n* = 4), Paecilomyces spp. (*n* = 2), Beauveria bassiana (*n* = 1), and Colletotrichum sp. (*n* = 1). Beauveria grew only at 28 °C, confined to the superficial corneal stroma. Isolates capable of growth up to 35 °C showed variable clinical courses, with two cases limited to the superficial stroma and one extending into the deep stroma. All Fusarium isolates grew at 37 °C, including two cases wherein the lesion extended through Descemet’s membrane and reached the anterior chamber, thus necessitating therapeutic keratoplasty.

**Conclusions:**

In this case series, isolates that grew only at 28 °C were restricted to the superficial corneal stroma, whereas those capable of growth at 37 °C were associated with deeper stromal or anterior chamber involvement. Growth temperature testing may therefore provide additional insight into the potential depth of lesion progression in filamentous fungal keratitis.

## Background

Infectious keratitis is a critical, vision-threatening corneal infection that can result in severe visual impairment and, in some cases, blindness. Fungal keratitis is particularly difficult to manage and is associated with poor visual outcomes. Its global burden is estimated to be more than one million cases per year [1]. Both filamentous fungi and yeast-like fungi can act as causative pathogens. Filamentous fungal keratitis often results from corneal trauma caused by plant material or by contact lens wear [2, 3], and various causative species have been reported, including *Fusarium* spp. and *Alternaria* spp. [4]. However, yeast-like fungal keratitis typically occurs in the setting of ocular surface disease or local ocular immunosuppression, such as topical corticosteroid use [5, 6], and is most often caused by *Candida* species [4].

Fungal keratitis is generally managed with a combination of topical and systemic antifungal agents plus surgical interventions such as therapeutic keratoplasty, but it is challenging to treat. Many filamentous fungi are poorly susceptible to antifungal agents. A multicenter study in Japan by Kimura et al. reported the in vitro susceptibility rates of filamentous fungi isolated from infectious keratitis, mostly revealing low susceptibility rate (micafungin: 18.2%, amphotericin B: 45.5%, fluconazole: 4.5%, voriconazole : 31.8%, miconazole: 18.2%, and pimaricin: 100%) [7]. While voriconazole penetrates corneal tissue well, it has a relatively low susceptibility rate against filamentous fungi [7–9]. Conversely, pimaricin exhibited excellent susceptibility against filamentous fungi, but it has poor corneal penetration [7, 10]. Therefore, if the focus of infection extends into the deep stroma, conservative treatment with antifungal agents alone is often insufficient, and therapeutic keratoplasty should be considered.

Given this background, we hypothesized that the optimal growth temperature of the fungi can contribute to the progression of filamentous fungal keratitis from the deep stroma into the anterior chamber. The corneal temperature is approximately 35 °C, while the anterior chamber temperature is approximately 37°C [11, 12]. Thus, the ability of a fungus to grow at these temperatures can influence the depth of corneal invasion. Accordingly, we retrospectively reviewed cases of fungal keratitis to investigate the relationship between fungal growth temperature and the depth of corneal infiltration.

## Methods

### Study design and clinical assessment

This single-center, retrospective study analyzed eight cases of filamentous fungal keratitis diagnosed at Ehime University Hospital between January 2020 and April 2024, wherein causative organisms were identified from corneal scrapings. We examined the clinical characteristics and the relationship between the fungal growth temperature and depth of corneal infiltration, as determined by growth temperature testing of the isolated strains. The depth of corneal stromal infiltration and the suspected extension of the lesion beyond Descemet’s membrane into the anterior chamber were clinically evaluated using slit-lamp examination and anterior segment optical coherence tomography (AS-OCT; Tomey Corporation, Nagoya, Japan), with particular attention to the presence of retrocorneal plaque formation [13].

### Identification of causative fungi

Fungal species were identified based on the morphological characteristics of slide cultures and giant colonies. Some isolates were identified via matrix-assisted laser desorption ionization time-of-flight mass spectrometry (MALDI-TOF MS; Bruker Daltonics, Billerica, MA) or sequence analysis of the rDNA internal transcribed spacer (ITS) region and the translation elongation factor 1α (TEF) region.

### Growth temperature testing

Growth temperature testing was performed using 6.1% Sabouraud agar plates prepared from powdered Sabouraud agar (EIKEN CHEMICAL CO., LTD, Tokyo, Japan). A fungal colony obtained via a platinum loop was inoculated vertically at the center of the agar plate. Each isolate was incubated aerobically (21% oxygen, ≤0.5% carbon dioxide) in separate plates of 28 °C, 35 °C, and 37°C°C, evaluating colony formation in each plate after 5 days of incubation.

### Ethics approval

This study was approved by the Ethics Committee of Ehime University (approval number 1,503,007) and conducted in accordance with the principles of the Declaration of Helsinki.

## Results

This case series included six males and two females with a mean age of 61.1 ± 22.5 years (Table 1). The identified fungal species were Fusarium spp. (*n* = 4), Paecilomyces spp. (*n* = 2), and Beauveria bassiana and Colletotrichum sp. (*n* = 1 each). The ocular predisposing factors were corneal trauma caused by plant material or contamination with soil or wastewater (*n* = 6), contact lens wear (*n* = 2), and topical corticosteroid use (*n* = 2). The systemic predisposing factors included diabetes mellitus (*n* = 2). Growth temperature testing revealed that Beauveria *bassiana* grew only at 28 °C, while Paecilomyces spp. and Colletotrichum sp. grew at both 28 °C and 35°C°C. Fusarium spp. grew at 28 °C, 35 °C, and 37°C°C. The growth temperatures varied depending on the fungal species. Correlating this with the depth of corneal infiltration, in the single case where the fungi grew only at 28 °C, its infiltration was confined to the superficial cornea throughout the clinical course. Meanwhile, in the three cases where the fungi grew up to 35 °C, the infiltration was limited to the superficial stroma in two cases and extended into the deep stroma in one case. All four cases capable of growth at 37 °C were Fusarium spp., and the fungal infiltrate penetrated Descemet’s membrane and reached the anterior chamber in two cases. All patients received combination topical antifungal therapy, and three additionally received systemic antifungals. Therapeutic keratoplasty was required in three cases, including two cases wherein the infection had extended into the anterior chamber. No recurrence of fungal infection or postoperative complications was observed following therapeutic keratoplasty in the three cases that required surgery. The duration of intensive topical antifungal therapy (≥6 times daily) tended to be longer in cases necessitating keratoplasty. In all cases, the final best-corrected visual acuity (BCVA) was either improved or comparable to the initial BCVA.

**Table 1:**
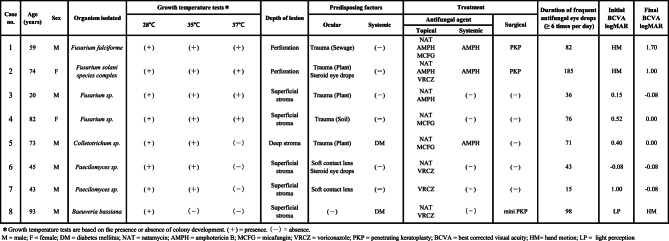
Characteristics of patients

### Representative cases

#### Case 2

**Fig. 1:**
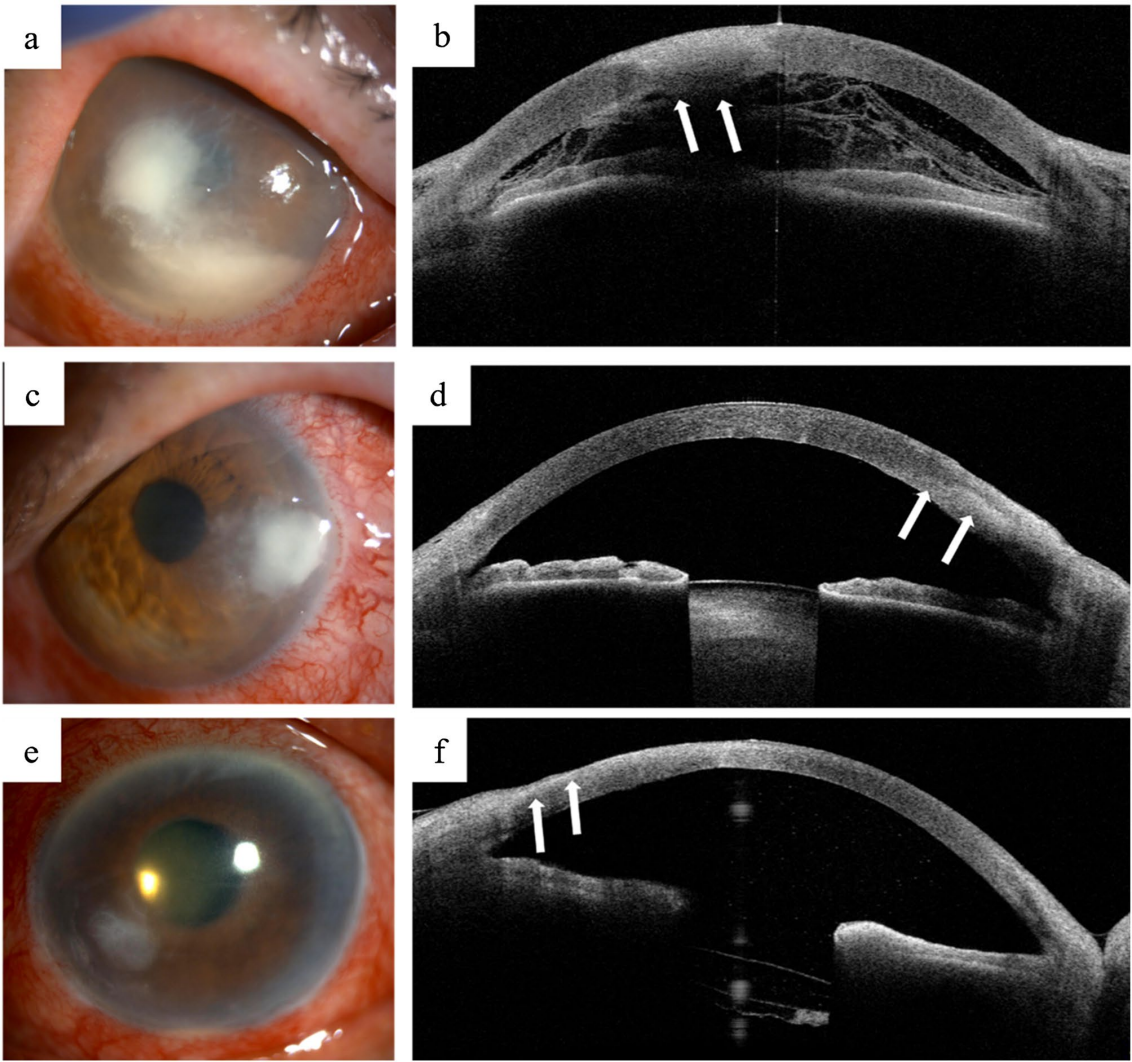
Slit-lamp photographs and anterior segment optical coherence tomography (AS-OCT) images of representative cases. (**a**), (**b**) Case 2: a paracentral corneal infiltrate extending through the full thickness of the stroma, with retrocorneal plaque formation indicating possible extension beyond Descemet’s membrane toward the anterior chamber (white arrows). (**c**), (**d**) Case 5: a temporal peripheral corneal infiltrate extending into the deep stroma but without anterior chamber involvement (white arrows). (**e**), (**f**) Case 8: an inferonasal corneal infiltrate confined to the superficial stroma (white arrows)

**Fig. 2:**
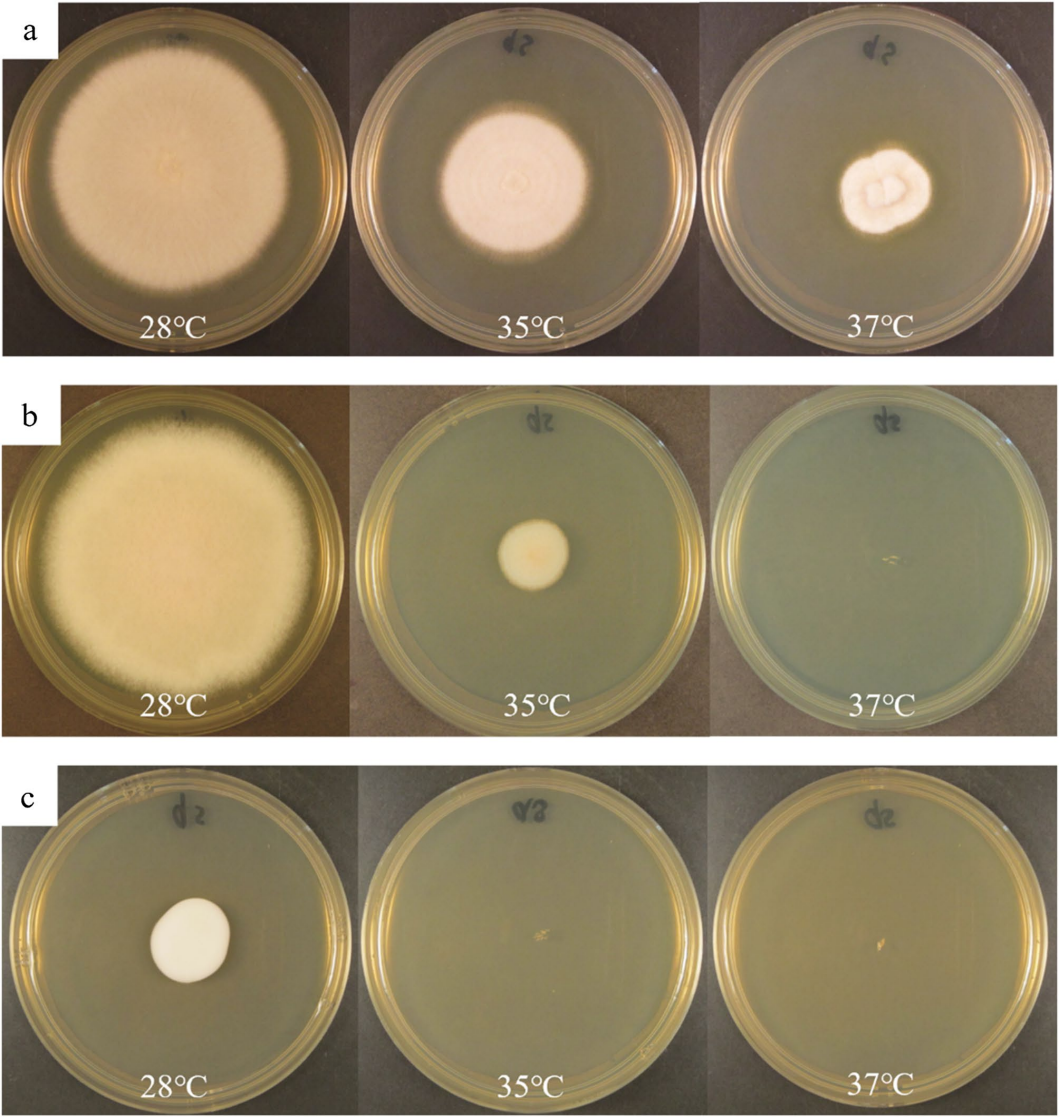
Photographs of colonies on agar plates from growth temperature testing. (**a**) Case 2: colony formation observed at all tested temperatures. (**b**) Case 5: colony formation observed at 28 °C and 35 °C but not at 37°C. (**c**) Case 8: colony formation observed only at 28 °C

A 74-year-old female developed a paracentral corneal infiltrate in the right eye while being treated for herpetic stromal keratitis. The infiltrate worsened despite treatment with topical 1% natamycin ointment, prompting referral to our hospital. Upon examination, the infectious lesion in the right cornea extended through the full thickness of the stroma. Based on retrocorneal plaque formation identified through slit-lamp examination and AS-OCT, the lesion was clinically considered to have extended beyond Descemet’s membrane into the anterior chamber (Fig. 1). Corneal scrapings revealed filamentous fungi on Gram staining, and MALDI-TOF MS of the cultured colonies identified the organism as *Fusarium solani* species complex. Growth temperature testing of the isolate revealed optimal growth at 28 °C, with additional growth at 35 °C and 37°C°C (Fig. 2). Treatment consisted of frequent topical administration of 5% natamycin, 0.1% amphotericin B, and 0.1% voriconazole, as well as 1% natamycin ointment and intravenous amphotericin B (100 mg/day). The infiltrate gradually decreased in size, although there was severe corneal thinning due to infection. Therapeutic penetrating keratoplasty was performed on day 44. The infection subsequently resolved without recurrence.

#### Case 5

A 73-year-old male with a history of diabetes mellitus sustained ocular trauma of the left eye by a mandarin tree branch. Due to persistent ocular pain and conjunctival hyperemia, he consulted at a local clinic 5 days later. Infectious keratitis of the left eye was diagnosed, prompting referral to our hospital. Upon examination, an oval-shaped corneal infiltrate was observed in the temporal peripheral cornea of the left eye. The infiltration extended into the deep stroma, but there was no retrocorneal plaque nor any evidence of anterior chamber involvement (Fig. 1). Corneal scrapings revealed filamentous fungi on Gram staining, and MALDI-TOF MS of the cultured colonies identified the organism as *Colletotrichum* sp. Growth temperature testing revealed optimal growth at 28 °C, slight colony growth at 35 °C, and no growth at 37 °C (Fig. 2). The patient was treated with frequent topical administration of 0.1% micafungin and 1% natamycin ointment. The corneal infiltrate gradually regressed, eventually achieving complete resolution. Although the fungus infiltrated the deep stroma, it did not penetrate Descemet’s membrane or reach the anterior chamber.

#### Case 8

A 93-year-old male consulted at a local clinic with pain in the left eye and was diagnosed with infectious keratitis, prompting referral to hospital. Upon examination, the left eye had a superficial paracentral corneal infiltrate accompanied by an epithelial defect and hypopyon, but without retrocorneal plaque formation (Fig. 1). Gram staining and culture of corneal scrapings from the lesion revealed filamentous fungi. However, species-level identification was not achieved despite morphology of the giant colonies and conidia alongside MALDI-TOF MS analysis. Subsequent sequencing of the ITS region identified the organism as *Beauveria bassiana*. Growth temperature testing demonstrated colony formation only at 28 °C, with no growth at 35 °C or 37 °C (Fig. 2). Treatment included frequent topical administration of 1% voriconazole and 0.1% micafungin, and 1% natamycin ointment. However, the infiltrate did not improve, with no significant effect observed on multiple corneal scrapings performed throughout the clinical course. Nevertheless, the infection focus remained confined to the superficial corneal stroma without progression to the deeper layers. On day 34, the lesion was removed via therapeutic penetrating keratoplasty, resulting in its complete resolution.

## Discussion

In this case series, we investigated the relationship between fungal growth temperature and the depth of corneal lesions in eight cases of filamentous fungal keratitis. Infectious keratitis caused by filamentous fungi is generally associated with trauma from plant material or similar sources, reflecting the natural habitats of these organisms. In this series, five of eight cases were associated with agricultural injuries or contamination with soil or wastewater, while predisposing factors such as corticosteroid use and diabetes mellitus were present in two cases each. Both cases of *Paecilomyces* spp. keratitis were associated with contact lens wear, which has also been identified as a major predisposing factor for *Paecilomyces* spp. keratitis in previous reports, suggesting its involvement in disease pathogenesis [14–18].

Growth temperature testing showed distinct patterns among the causative fungi. Beauveria bassiana grew only at 28 °C, whereas *Paecilomyces* spp. and *Colletotrichum* sp. grew at 28 °C and 35°C°C but not at 37 °C. In contrast, all *Fusarium* spp. isolates grew at all tested temperatures, including 37 °C. Clinically, the lesion caused by the isolate that grew only at 28 °C remained confined to the superficial stroma, whereas some *Fusarium* cases caused by isolates capable of growth at 37 °C showed deep stromal or anterior chamber involvement. These findings suggest that fungal growth temperature may be associated with the depth of corneal lesions. However, the clinical course could not be explained by growth temperature alone, particularly in Cases 3, 4, and 5. Therefore, the results should be interpreted as indicating that growth temperature is one of several factors that may contribute to lesion progression, rather than the sole determinant of lesion depth.

Case 8 was caused by *Beauveria bassiana*, which grew only at 28 °C and was confined to the superficial cornea throughout the course of the disease. The therapeutic response was limited despite frequent topical administration of antifungal agents, and repeated microscopic examinations of corneal scrapings consistently revealed filamentous fungi. Ultimately, therapeutic penetrating keratoplasty was required, but in the 35 days prior to surgery, the lesion did not progress into the deep stroma. Consistent with these observations, Mitani et al. reported a case of *B. bassiana* keratitis in which the lesion remained confined to the superficial cornea, and the isolate grew only at 28°C [19]. Similarly, Sonoyama et al. described a case of *B. bassiana* keratomycosis restricted to the superficial stromal layer [20]. Taken together, these findings suggest that keratitis caused by *B. bassiana* tends to remain relatively superficial. The inability of *B. bassiana* to grow at higher temperatures may partly explain why the lesion did not extend into deeper, warmer regions of the cornea or toward the anterior chamber.

All four cases of *Fusarium* keratitis demonstrated growth at all tested temperatures, including 37 °C. This finding indicates that these isolates were capable of proliferating under temperature conditions comparable to those in the deep cornea and anterior chamber. In fact, in Cases 1 and 2, the lesions were clinically considered to have extended from the deep cornea toward the anterior chamber, and therapeutic keratoplasty was required. Thus, the ability to grow at 37 °C may have been associated with deep lesion progression in at least some *Fusarium* cases.

Cases 3 and 4 were also caused by *Fusarium* isolates capable of growth at 37 °C, but the lesions remained confined to the superficial stroma. Thus, growth at 37 °C alone cannot fully account for the differences in lesion progression among the *Fusarium* cases. Several additional factors may have contributed to these differences. In the two *Fusarium* cases in which the lesions remained limited to the superficial stroma (Cases 3 and 4), antifungal treatment was initiated 6 and 10 days after symptom onset, respectively. By contrast, in the cases in which the lesions penetrated Descemet’s membrane and extended toward the anterior chamber (Cases 1 and 2), antifungal therapy was initiated after 10 and 30 days, respectively. Earlier initiation of antifungal therapy may have limited progression into the deep stroma or anterior chamber. In addition, although antifungal susceptibility testing was not performed in this study, differences in antifungal susceptibility may have influenced treatment outcomes and the depth of corneal invasion. Todokoro et al. reported variability in *Fusarium* susceptibility, including relatively high minimum inhibitory concentrations for voriconazole [21]. Furthermore, a previous clinical study reported that *Fusarium* keratitis can be caused by diverse *Fusarium* species or species complexes, with corresponding variability in antifungal susceptibility and clinical outcomes [22].

Case 5 involved *Colletotrichum* sp., which grew at 28 °C, showed slight growth at 35 °C, but failed to grow at 37 °C. In this case, the lesion extended into the deep stroma but did not exhibit retrocorneal plaque formation or anterior chamber involvement. Shiraishi et al. previously reported three cases of *Colletotrichum gloeosporioides* keratitis in which lesions were confined to the anterior stroma without deep stromal extension [23]. In that study, all isolates grew well at 25 °C but poorly at 35 °C and 37°C°C, suggesting that reduced growth at higher temperatures may be associated with more superficial lesion localization. In the present Case 5, however, the lesion reached the deep stroma despite the absence of growth at 37 °C. This inconsistency may be explained by the initial depth of fungal inoculation following plant-related trauma; the causative fungus may have been introduced directly into the deep stroma at the time of injury. On the other hand, the absence of growth at 37 °C may be related to the absence of anterior chamber involvement.

Taken together, the depth of lesion progression in filamentous fungal keratitis should be interpreted as the result of multiple interacting host-, treatment-, and pathogen-related factors rather than fungal growth temperature alone. These factors may include species-specific fungal pathogenicity, antifungal susceptibility, timing of antifungal therapy, initial depth of traumatic inoculation into the cornea, prior topical corticosteroid use, and host factors such as diabetes mellitus. In the present study, topical corticosteroids were used during the clinical course in two cases (Cases 2 and 6). Prior topical corticosteroid use in Case 2 (*Fusarium solani* species complex) may have contributed to lesion progression because corticosteroids can suppress local immune defenses and thereby potentially exacerbate fungal infection. In Case 6 (*Paecilomyces* sp.), discontinuing the corticosteroid eye drops did not worsen the infectious lesion or inflammatory findings, and the stromal infiltrate did not reach the deep cornea. Hübner et al. reported cases of contact lens–associated *Paecilomyces lilacinus* keratitis that presented as superficial stromal infiltrates without progression into deeper corneal layers, a clinical course that aligns in part with the findings observed in Case 6 [18].

Host inflammatory responses may also play an important role in determining the severity and progression of fungal keratitis. In a murine *Aspergillus fumigatus* keratitis model, Lin et al. demonstrated that punicalagin not only inhibited fungal processes such as hyphal growth and biofilm formation, but also attenuated host responses—including macrophage infiltration, inflammatory cytokine release, and reactive oxygen species generation—ultimately reducing the clinical severity of keratitis [24]. These findings highlight that the clinical course of fungal keratitis is determined not only by fungal proliferative capacity but also by the magnitude of the host inflammatory response.

In this case series, final BCVA in all cases generally improved compared with baseline; however, three patients required therapeutic keratoplasty during the clinical course. In two of these cases, the causative fungi demonstrated growth at 37 °C, and the lesions were clinically judged—based on AS-OCT and slit-lamp findings—to have extended from the deep cornea into the anterior chamber. In such instances, disease control with conservative antifungal therapy alone proved challenging, necessitating keratoplasty. These observations suggest that growth temperature testing of causative fungi may provide supplementary information for estimating the risk of deep corneal progression and could be valuable when considering therapeutic keratoplasty during management. However, such information should be interpreted in conjunction with other clinical and microbiological factors and should not be used in isolation.

This study has several limitations. First, this was a single-center study with a small number of cases. Further studies with larger cohorts are needed to clarify the relationship between growth temperature and the depth of corneal infection in fungal keratitis. Second, the depth of corneal involvement was assessed using slit-lamp examination and AS-OCT. While these modalities are valuable for clinical evaluation, definitive assessment of fungal invasion depth would require histopathological and microbiological confirmation. Thus, the possibility of misclassification of lesion depth cannot be completely excluded and should be addressed in future research. Third, in trauma-related keratitis, the depth of the initial injury may be an important determinant of subsequent lesion progression; however, this factor could not be evaluated in the present study. Fourth,　the inoculum size used for growth temperature testing was not strictly standardized, as quantitative standardization of filamentous fungi is inherently difficult. Moreover, colony formation was assessed after a single incubation period of 5 days. Further in vitro and in vivo studies employing standardized inoculum sizes and varied incubation conditions are needed to clarify the reliability and clinical significance of growth temperature testing. Finally, although the temperature settings in this study were based on previous reports of corneal and anterior chamber temperatures, the actual temperature difference within the cornea was not directly measured. Consequently, the impact of the approximately 2 °C temperature difference between these anatomical sites on fungal proliferation and lesion development within the cornea remains uncertain. Therefore, fungal growth temperature should be considered one potential contributing factor to lesion depth rather than a definitive determinant of lesion progression.

## Conclusions

This case series investigated the clinical features of filamentous fungal keratitis and explored the relationship between the depth of corneal stromal infiltration and the growth temperature of the causative fungi. Isolates capable of growth only at 28 °C tended to remain confined to the superficial corneal stroma, whereas those able to grow at 35 °C or 37 °C demonstrated variable depths of stromal invasion. Although isolates capable of growth at 37 °C included cases with deep stromal or anterior chamber involvement, lesion progression was likely influenced by multiple factors—including fungal species, antifungal susceptibility, timing of therapy, depth of traumatic inoculation, corticosteroid exposure, and host-related factors. Taken together, these findings suggest that growth temperature testing may provide supplementary information for estimating the risk of deep corneal progression in filamentous fungal keratitis.

## Data Availability

The datasets generated and/or analyzed during the current study are available from the corresponding author on reasonable request.

## Abbreviations

AS-OCT: anterior segment optical coherence tomography
MALDI-TOF MS: matrix-assisted laser desorption ionization time-of-flight mass spectrometry
ITS: internal transcribed spacer
TEF: translation elongation factor 1α
BCVA: best-corrected visual acuity
